# Supplementary material for: ACE2 Receptor and Its Isoform Short-ACE2 Are Expressed on Human Spermatozoa
Source: Int J Mol Sci. 2022 Mar 28;23(7):3694. doi: 10.3390/ijms23073694 (PMC8998905; doi:10.3390/ijms23073694)
Supplement: Supplementary file 1 [file ijms-23-03694-s001.zip › ijms-1627388-supplementary.pdf]

Supplementary information

A)

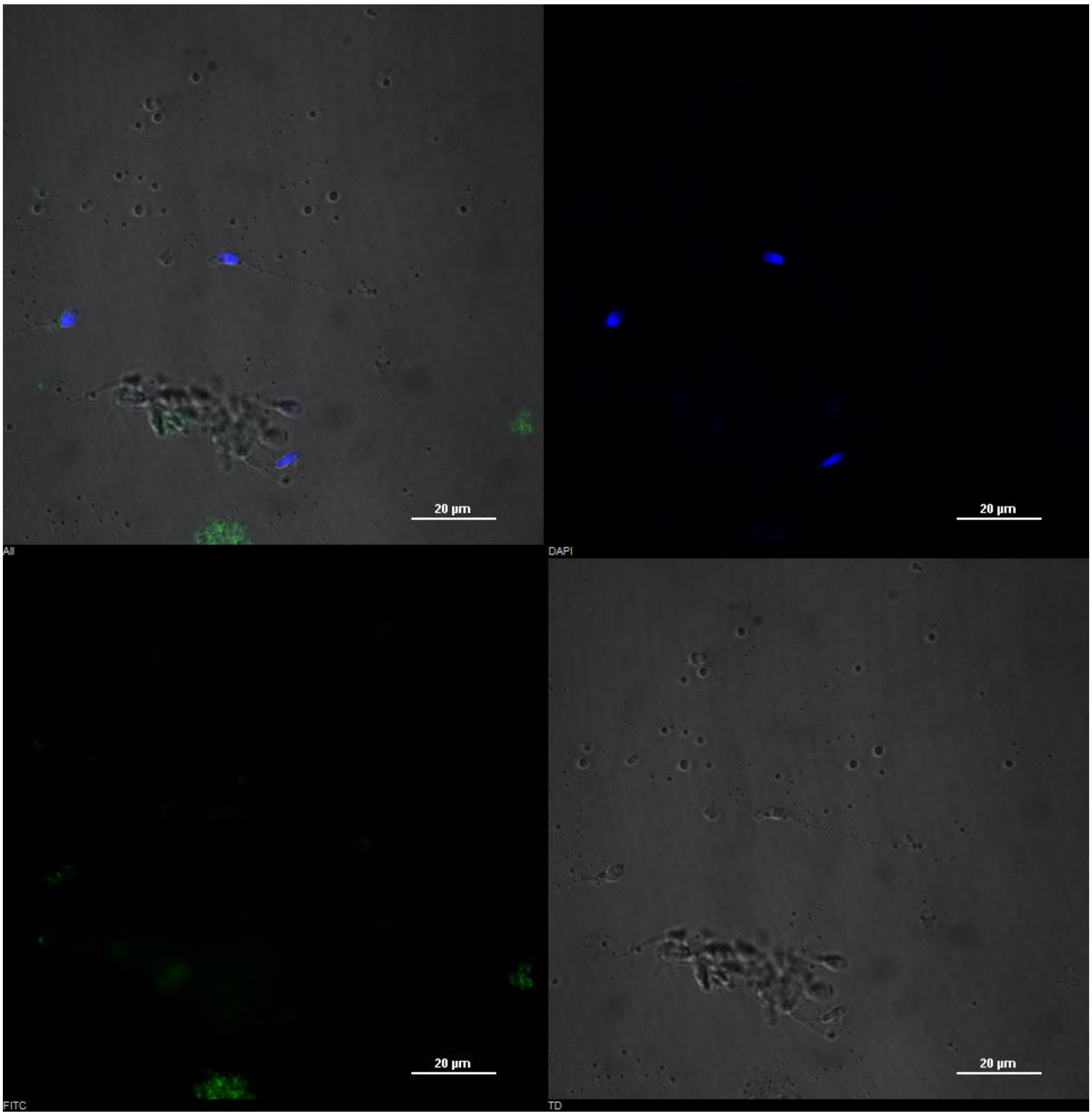

B)

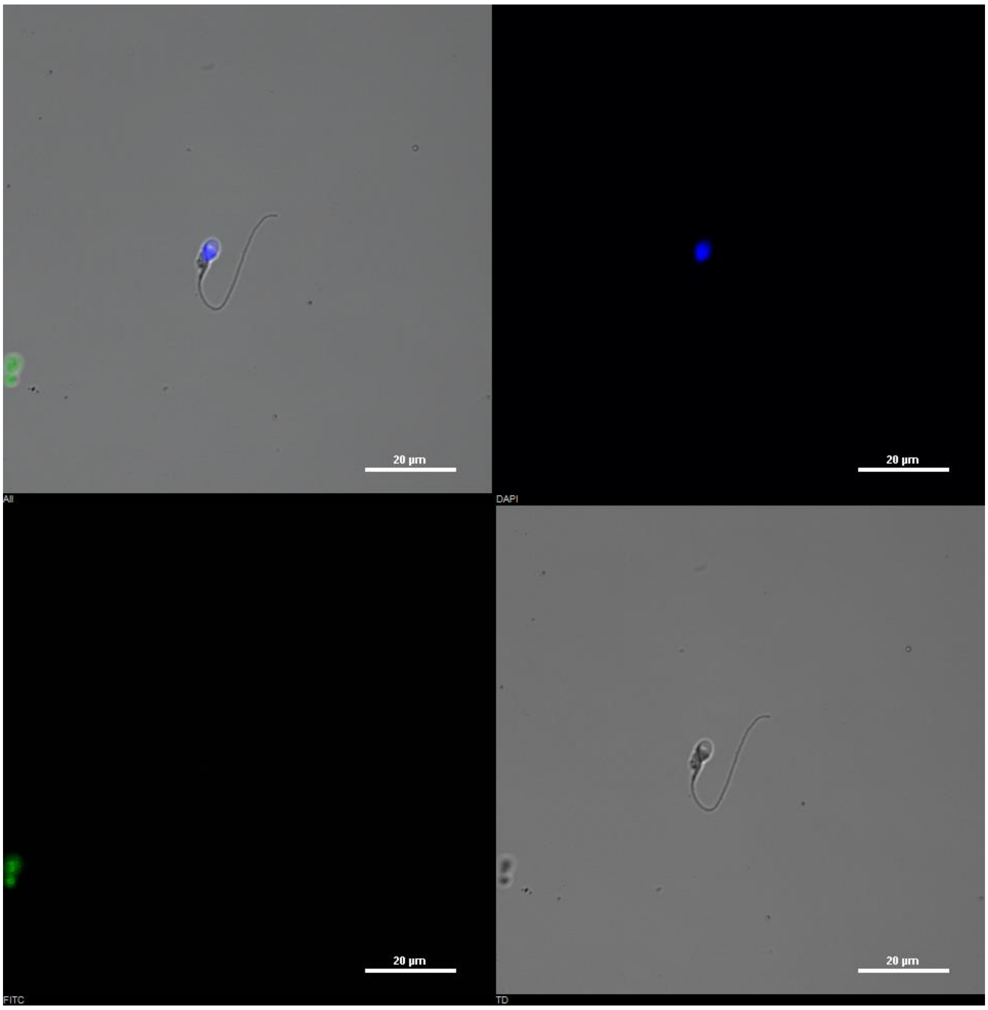

Supplementary figure S1. A) Negative control for immunocytochemistry assays, avoiding the use of the primary antibody and using anti-rabbit secondary antibodies. B) Representative image of a negative control for immunocytochemistry assays, performed using rabbit serums instead of the primary antibody, followed by incubation with the secondary antibody.

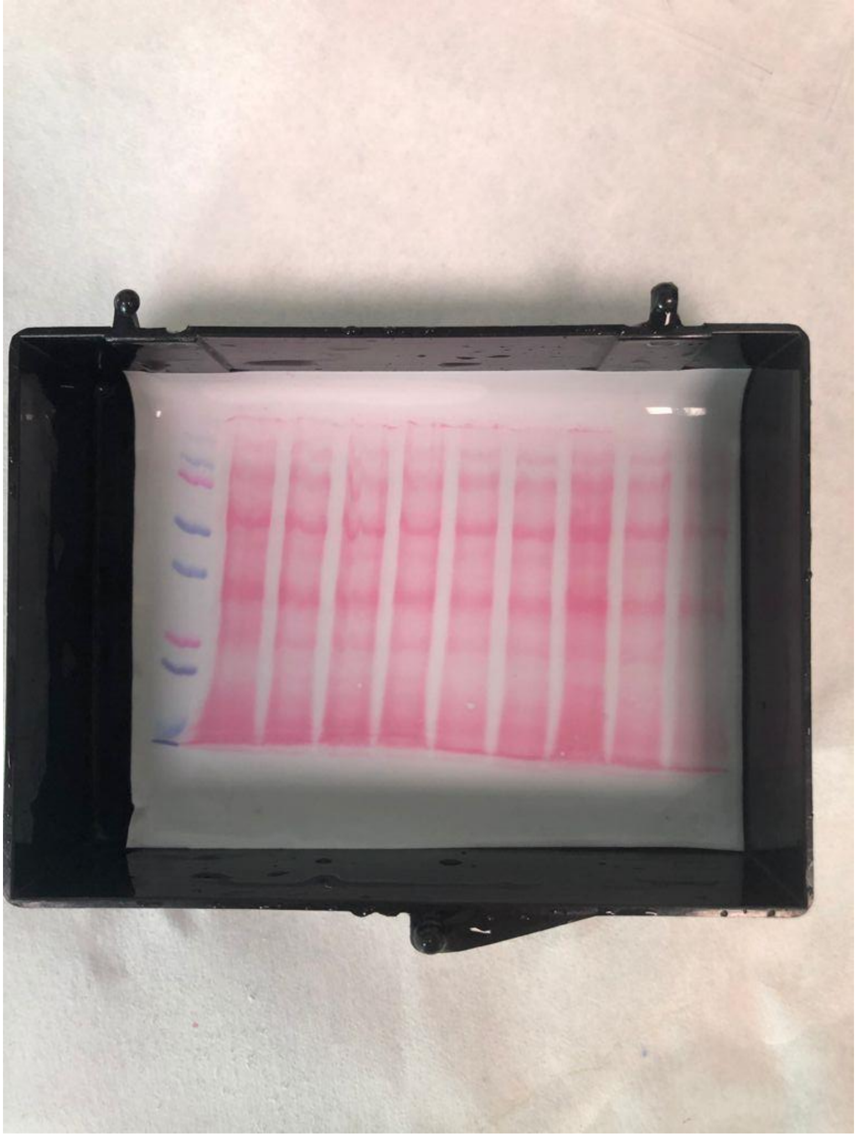

Supplementary figure S2. Representative image of a membrane prior to hybridization. Blots used were mini size. This figure shows a membrane after Ponceau staining and prior to the incubation with the corresponding antibody.

A)

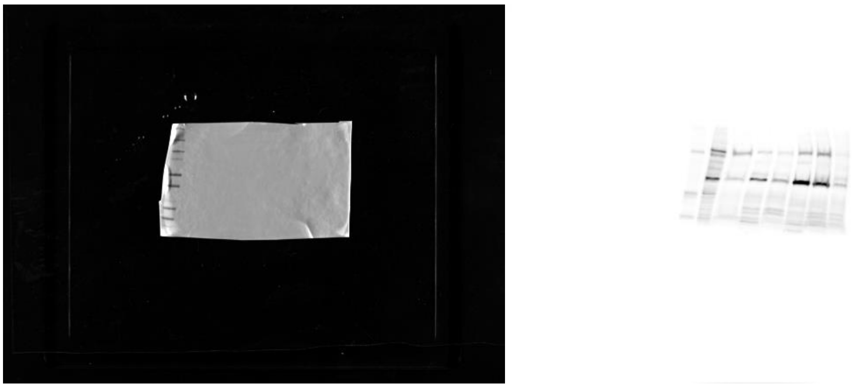

B)

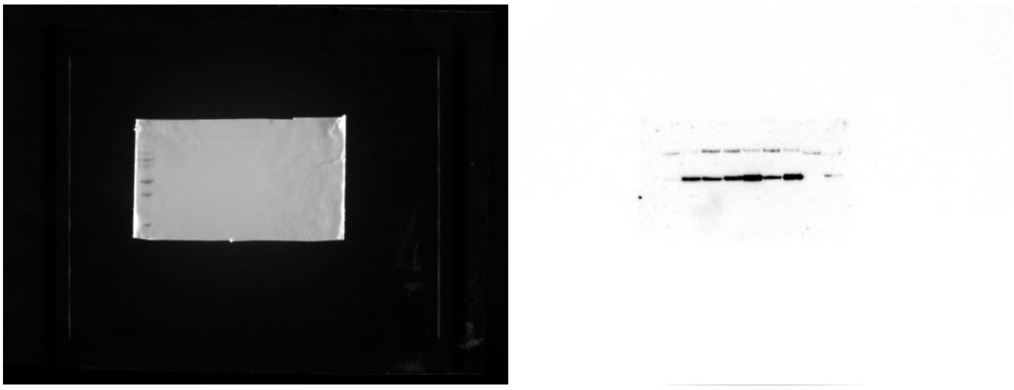

C)

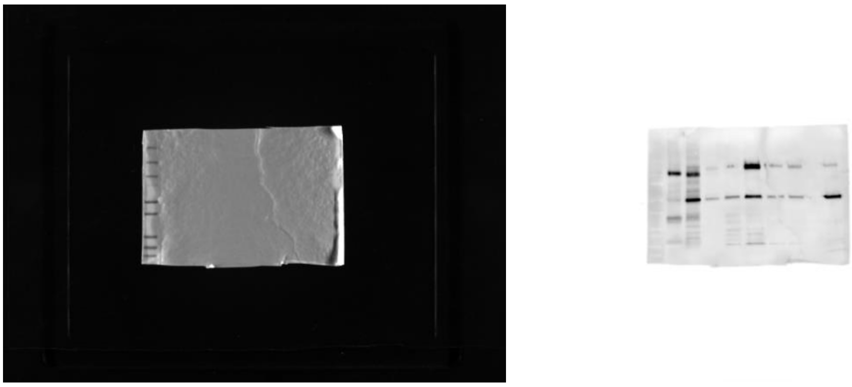

Supplementary figure S3. Three representative blots with full-length membranes and membrane edges for anti-ACE2-1 (abcam). Left: molecular weight marker; right: immunoblotting.

A)

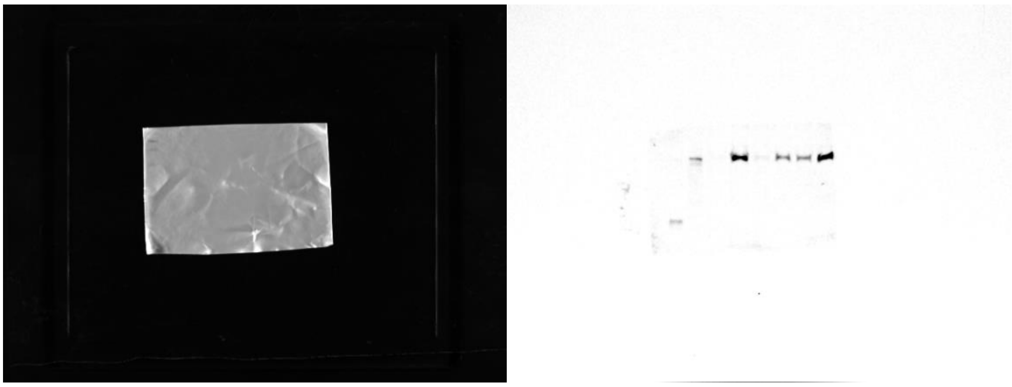

B)

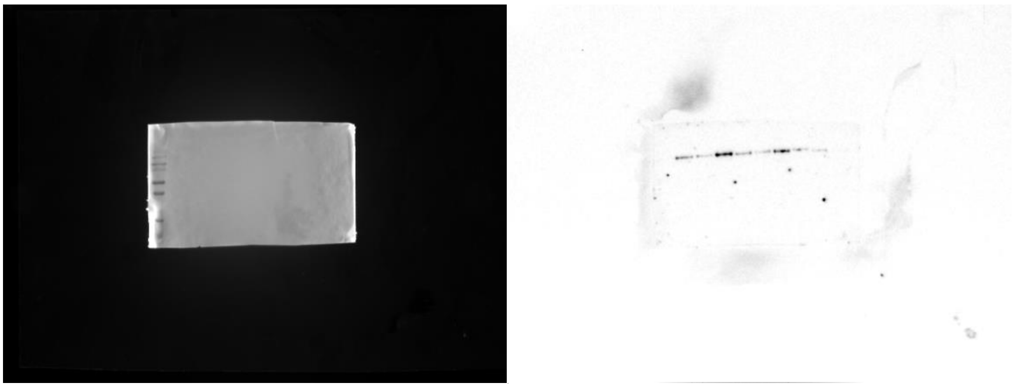

C)

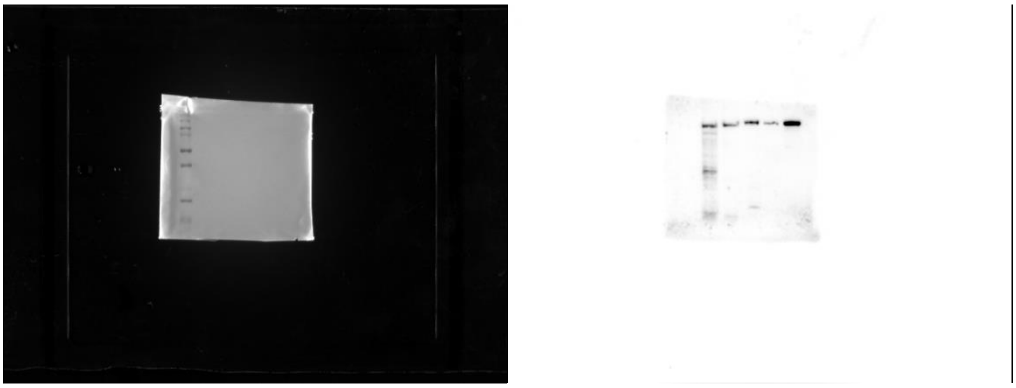

Supplementary figure S4. Three representative blots with full-length membranes and membrane edges for anti-ACE2-2 (Novus). Left: molecular weight marker; right: immunoblotting.

**Semen parameters**	**Mean ± standard error**
Semen volume (ml)	3.20 ± 0.24
Sperm concentration (x10^6/ml)	51.80 ± 5.39
Progressive motility (%)	49.15 ± 2.06
Total motility (%)	64.75 ± 2.07
Normal morphology (%)	15.75 ± 1.36

Supplementary Table S1. Semen parameters of the whole study population (N=40 healthy donors).
